# Factors Associated With the Success of External Cephalic Version for Breech Presentation: A Retrospective Study

**DOI:** 10.7759/cureus.87413

**Published:** 2025-07-07

**Authors:** Yuri Hasegawa, Chiaki Eishi, Koh Nagata, Ai Nagata, Shoko Miura, Kiyonori Miura

**Affiliations:** 1 Obstetrics and Gynecology, Nagasaki University Graduate School of Biomedical Sciences, Nagasaki, JPN

**Keywords:** breech presentation, external cephalic version, factors for ecv success, fetal buttock engagement, nuchal cord

## Abstract

Objective

The aim of this study is to retrospectively evaluate additional factors potentially associated with the success of external cephalic version (ECV), including fetal buttock engagement in the maternal pelvis and umbilical cord entanglement, in addition to multiparity, th absence of maternal obesity, a posterior placental location, an estimated fetal body weight > 2500 g, adequate amniotic fluid, and the use of tocolytic agents or anesthesia.

Methods

This retrospective study included women with singleton pregnancies who underwent ECV at our institution between January 2011 and December 2024. Women with an abnormal placental location, fetal growth restriction, prior cesarean delivery, or missing data were excluded. Maternal background and ultrasound findings, such as maternal age, parity, body mass index, gestational age at ECV, presence of uterine contractions, use of tocolytic agents, estimated fetal body weight, fetal buttock engagement (the presence of an amniotic fluid pocket between the fetal buttocks and the internal cervical os), maximum vertical pocket, placental location, and a nuchal cord, were reviewed. A logistic regression analysis was performed to identify independent predictors of the success of ECV.

Results

Among 69 women who had ECV, 59 were included in the analysis after excluding those with missing data. The ECV success rate was 57.6%. Multiparity (odds ratio (OR): 1.53, 95% confidence interval (CI): 0.49-4.79) and use of tocolytic agents (OR: 3.53, 95% CI: 0.46-24.4) were associated with a trend toward higher success rate of ECV. The absence of fetal buttock engagement (OR: 24.7, 95% CI: 3.61-168.7, p = 0.001) and the absence of a nuchal cord (OR: 4.75, 95% CI: 0.93-24.3, p = 0.006) were significantly associated with successful ECV.

Conclusion

In addition to previously known factors, the absence of fetal buttock engagement and the absence of a nuchal cord were independently associated with successful ECV. These findings may help improve pre-procedural counseling and selection of candidates for ECV.

## Introduction

Breech presentation occurs in approximately 3%-4% of all pregnancies [1]. In Japan, the rate of vaginal delivery for breech presentation has been decreasing in recent years, while the cesarean section rate continues to rise. External cephalic version (ECV) is a procedure that converts breech presentation to cephalic presentation, increasing the possibility of vaginal delivery and reducing the cesarean rate. Despite the potential benefits of ECV, it remains underused, with reports indicating that 20%-30% of eligible women in Western countries do not undergo this procedure [2,3]. Previous studies have identified factors associated with successful ECV, such as multiparity, the absence of maternal obesity, a posterior placental location, an estimated fetal body weight (EFBW) > 2500 g, adequate amniotic fluid, and the use of tocolytic agents or anesthesia [4]. This study aimed to retrospectively evaluate the effect of fetal buttock engagement and a nuchal cord, in addition to established factors, on the success of ECV performed under a standardized protocol at our institution.

## Materials and methods

ECV protocol

ECV was conducted with written informed consent. The following criteria were selected as prerequisites for performing the ECV protocol. Before initiating ECV, the mother was placed in a head-down position. Cardiotocography was performed, and the reassuring fetal status was confirmed. Uterine contraction inhibitors were administered by intravenous infusion, and ECV was initiated after at least 30 minutes had elapsed. ECV was performed by an obstetrician with experience in at least 50 cases or under the supervision of such a physician. Participants who met all selection criteria and did not meet any exclusion criteria were included in the study. The selection criteria were as follows: 1) breech or transverse presentation, 2) singleton pregnancy, and 3) gestational age of 36 weeks and 0 days or more. The exclusion criteria were as follows: 1) abnormal placental location, 2) fetal growth restriction, and 3) previous cesarean section. If transient fetal bradycardia occurred, the procedure was temporarily suspended, and the fetal heart rate was allowed to recover. ECV was resumed once bradycardia resolved. If bradycardia reoccurred, the ECV was terminated. After terminating ECV, the reassuring fetal status was confirmed by cardiotocography, regardless of success or failure.

ECV procedure

Gel was applied to the pregnant woman’s abdomen to reduce friction. After confirming the fetal position and placental location using abdominal ultrasound, the operator’s hands were placed on the pubic bone, and the fetal buttocks were lifted. If the buttocks did not lift, the procedure was discontinued. Once the buttocks were lifted from the mother’s pelvis, the fetal buttocks were pushed upward to rotate the fetus forward. The fetal head was pushed downward in the opposite direction of the buttocks to rotate the fetus. An abdominal ultrasound device was used as needed to confirm the fetal heartbeat.

ECV targets and considerations

We reviewed cases of women with singleton pregnancies who underwent ECV at our hospital between January 2011 and December 2024. Patients with incomplete data were excluded.

Maternal characteristics and ultrasound findings, including maternal age, parity, body mass index at ECV, gestational age at ECV, presence of uterine contractions (evaluated via 40-minute cardiotocography), and use of tocolytic agents, were retrospectively collected. Ultrasound findings included EFBW, the presence or absence of fetal buttock engagement in the pelvis, the maximum vertical pocket, placental location, and a nuchal cord. Fetal buttock engagement was defined as the presence of an amniotic fluid pocket between the fetal buttocks and the internal cervical os. A nuchal cord was defined as one or more complete loops of the cord around the fetal neck. This study assessed umbilical cord entanglement using transabdominal ultrasonography, but limited the analysis to nuchal cord, excluding cases involving the fetal trunk or extremities.

Statistical analysis

A logistic regression analysis was performed to assess factors associated with successful ECV. Statistical analyses were performed using JMP Pro 16 (version 16.2.0; SAS Institute Inc., Cary, NC, USA). A P-value of 0.05 was used as the threshold for a significant difference.

## Results

Maternal characteristics and ultrasound findings

During the study period, 69 women underwent ECV, and 59 were included in the final analysis. Maternal characteristics and ultrasound findings are shown in Table 1. The mean maternal age, height, weight, and body mass index were 31.2 (standard deviation: 4.9) years, 159.1 (5.2) cm, 60.7 (7.5) kg, and 23.9 (2.8) kg/m², respectively. Nulliparous women accounted for 59.3% (n = 35). Adverse events occurred in seven (11.9%) women, and the most common was transient fetal bradycardia (n = 6). The ECV success rate was 57.6% (n = 34). Among unsuccessful cases of ECV, the most frequent reason was fetal buttock engagement that prevented mobilization (64%), followed by fetal bradycardia leading to discontinuation (12%). One of these women required emergency cesarean section because of persistent bradycardia. The mean EFBW was 2475.0 (253.5) g. An anterior placental location and a nuchal cord were each observed in 17 (27.9%) women. ECV was performed at ≥ 37 weeks in 13.1% of women.

**Table 1:** Maternal background, ultrasound findings, and ECV implementation status. The table summarizes maternal background, ultrasound findings, and ECV implementation status. The mean maternal age was 31.3 years, and 59.3% were nulliparous. Mean height and weight were 159.1 cm and 60.7 kg, respectively, with a BMI of 23.9 kg/m². The mean estimated fetal body weight was 2,475.0 g, and the mean maximum vertical pocket was 4.6 cm. Anterior placental location was observed in 27.9%, nuchal cord in 27.9%, and fetal rump engagement in 25.4%. ECV was performed at ≥37 weeks in 13.6% of cases, with a success rate of 57.6%. Transient fetal bradycardia occurred in 10.2%, while placental hematoma and emergency cesarean section each occurred in 1.7%. No cases of vaginal bleeding were reported. ECV: external cephalic version, BMI: body mass index, EFBW: estimated fetal body weight, MVP: maximum vertical pocket.

	Mean (standard deviation) or n (%)	Range
Maternal background		
Age (years)	31.3 (4.9)	18-43
Nulliparity (%)	35 (59.3%)	
Height (cm)	159.1 (5.2)	146.9-170.0
Weight (g)	60.7 (7.5)	42-79
BMI (kg/cm^2^)	23.9 (2.8)	19.5-32.9
Ultrasound findings		
EFBW (g)	2,475.0 (253.5)	2,036-3,440
MVP (cm)	4.6 (1.4)	1.8-8.9
Placental position		
Anterior	17 (27.9)	
Non-anterior	42 (72.1)	
Presence of nuchal code	17 (27.9)	
Engagement of the fetal rump in the maternal pelvis	15 (25.4)	
ECV implementation status		
ECV performed at 37 weeks 0 days of pregnancy or later	8 (13.6)	
ECV success	34 (57.6)	
Complication during ECV		
Transient fetal bradycardia	6 (10.2)	
Vaginal bleeding	0 (0)	
Placental hematoma	1 (1.7)	
Emergency cesarean section	1 (1.7)	

Multivariable logistic regression of maternal characteristics and successful ECV

Multivariable logistic regression showed no significant associations between successful ECV and maternal factors (age < 35 years, multiparity, body mass index < 25 kg/m^2^, gestational age < 37 weeks, absence of uterine contractions, and use of tocolytic agents) (Table 2). However, a tendency toward a higher success rate of ECV was observed for multiparity and tocolytic use (odds ratio (OR): 1.53, 95% confidence interval (CI): 0.49-4.79 and OR: 3.53, 95% CI: 0.46-24.4, respectively) (Table 2).

**Table 2:** Multivariable logistic regression of maternal characteristics and successful ECV (n = 59). *No significant association was observed between maternal characteristics and the success of ECV. ECV: external cephalic version, BMI: body mass index, NS: not significant.

	Odds ratio	P-value*
Maternal age (years) <35	1.04 (0.29-3.67)	NS
Multipara	1.53 (0.49-4.79)	NS
BMI <25	0.48 (0.15-1.52)	NS
Gestational week at ECV (weeks) <37	0.31 (0.04-2.22)	NS
Non-uterus contraction	0.72 (0.12-4.54)	NS
Using tocolytic agent	3.53 (0.46-24.4)	NS

Multivariable logistic regression of ultrasound factors and successful ECV

Among ultrasound factors, the absence of fetal buttock engagement (OR: 24.7, 95% CI: 3.61-168.7, p = 0.001) and the absence of a nuchal cord (OR: 13.1, 95% CI: 2.07-82.7, p = 0.006) were significantly associated with the success of ECV (Table 3). Trends for an association with successful ECV were also observed for an EFBW > 2500 g (OR: 2.37, 95% CI: 0.50-11.3) and a non-anterior placental location (OR: 4.75, 95% CI: 0.93-24.3), although these did not reach significance (Table 3).

**Table 3:** Multivariable logistic regression of ultrasound factors and successful ECV (n = 59). Among the ultrasound factors, the absence of a nuchal cord and the absence of fetal rump engagement in the maternal pelvis were significantly associated with successful ECV. EFBW: estimated fetal body weight, MVP: maximum vertical pocket, NS: not significant, ECV: external cephalic version.

	Odds ratio	P-value
EFBW>2,500g	2.37 (0.50-11.3)	NS
MVP >4cm	0.53 (0.77-3.66)	NS
Non-anterior placental position	4.75 (0.93-24.3)	NS
No nuchal cord	13.1 (2.07-82.7)	0.006
No fetal rump engagement in the maternal pelvis	24.7 (3.61-168.7)	0.001

## Discussion

We performed a multivariate analysis of the absence of fetal buttock engagement and a nuchal cord in addition to previously reported factors for successful ECV [4,5]. This study showed that the absence of fetal buttock engagement and a nuchal cord were significantly associated with the success of ECV.

Factors found to be involved in successful ECV

While the association between fetal engagement and the failure of ECV may be intuitive, the ultrasound finding that non-engagement (presence of an amniotic fluid pocket between the fetal buttocks and maternal pelvis) predicts success has clinical utility for pre-procedural assessment. Few studies have investigated the role of the condition of the umbilical cord in outcomes of ECV [6]. Some studies have reported that cord length, but not entanglement, is associated with success, while others excluded cases of a nuchal cord [7]. Further investigation is required to clarify the clinical relevance of cord entanglement in ECV.

Although previously reported predictors, such as multiparity and the use of tocolytics, did not show significance in this study, a trend toward higher successful ECV was observed. A recent systematic review [5] supported the use of neuraxial analgesia in combination with tocolytics to improve the success of ECV. While neuraxial anesthesia was not used in our protocol, it may be considered in efforts to improve outcomes. ECV is generally safe, with reported risks of placental abruption, cord prolapse, rupture of membranes, stillbirth, and fetomaternal hemorrhage occurring in < 1% of cases [8,9]. Rare events, such as fetal death [10] or emergent cesarean due to placental abruption [11], emphasize the importance of conducting ECV in facilities capable of rapid surgical intervention.

Limitations of this study and policy for successful ECV

Limitations of our study include its retrospective design and small sample size. However, strengths of this study include a standardized protocol and procedures conducted by experienced obstetricians. Although the success of ECV does not always translate into a lower cesarean rate, numerous studies have shown that successful ECV increases the likelihood of vaginal delivery [12]. In our cohort, many deliveries occurred at community hospitals, limiting our ability to assess final delivery outcomes.

Based on previous reports on the success of ECV and our study, we recommend the following management policy for performing ECV. Prior to performing ECV, an abdominal ultrasound examination should be performed to confirm the presence of an amniotic fluid cavity between the fetal buttocks and the maternal pelvis, the absence of a nuchal cord, and the placental location (anterior or otherwise). Before performing ECV, uterine contraction inhibitors should be administered. By implementing these measures, we believe that ECV can be performed more safely.

## Conclusions

ECV is a safe and effective technique to reduce the cesarean delivery rate and should be offered more widely. Understanding the factors associated with successful ECV may enhance safety and improve the appropriate selection of patients.

In addition to established predictors, this study shows that the absence of fetal buttock engagement and the absence of a nuchal cord contribute to the success of ECV.
